# Was femoral nerve block effective for pain control of medial opening-wedge high tibial osteotomy? A single blinded randomized controlled study

**DOI:** 10.1097/MD.0000000000023978

**Published:** 2021-01-22

**Authors:** Yi-Ming Ren, Meng-Qiang Tian, Yuan-Hui Duan, Yun-Bo Sun, Tao Yang, Wei-Yu Hou, Shu-Hua Xie

**Affiliations:** a Department of Joint and Sport Medicine, Tianjin Union Medical Center, PR China; b Department of Anesthesiology, Tianjin Union Medical Center, PR China.

**Keywords:** analgesia, femoral nerve, high tibial osteotomy, nerve block, pain management

## Abstract

**Background and Purpose:**

Medial compartment femoro–tibial osteoarthritis (OA) is a common disease and opening-wedge high tibial osteotomy (OWHTO) is the common surgical procedure carried out for these patients. While most researchers are focusing on the surgical techniques during operation, the aim of this study is to evaluate the pain control effect of femoral nerve block (FNB) for OWHTO patients.

**Methods:**

In this prospective, single-center, randomized controlled trial (RCT) study, 41 patients were operated on by OWHTO for OA during 2017 to 2018. Twenty of them (group A) accepted epidural anesthesia with FNB and 21 patients (group B) only had their single epidural anesthesia. All blocks were successful and all the 41 patients recruited were included in the analysis and there was no loss to follow-up or withdrawal. Systematic records of visual analog scores (VAS), quadriceps strength, mean number of times of patient-controlled intravenous analgesia (PCIA), using of additional opioids or nonsteroidal anti-inflammatory drugs (NSAIDs), and complications were done after hospitalization. The Student *t* test and Chi-Squared test was used and all *P* values ≤.05 were considered statistically significant.

**Results:**

VAS scores at rest (3.48 ± 1.0 vs 4.68 ± 1.1) and on movemment (4.51 ± 0.6 vs 4.97 ± 0.8) decreased more in group A than group B with significance at follow-up of 12 hours. The quadriceps strength, consumption of additional opioids or NSAID injections and mean number of times that the patients pushed the PCIA button didnot differ significantly within each group.

**Conclusion:**

This RCT study shows that FNB in patients undergoing OWHTO for unicompartmental osteoarthritis of the knee could result in significant reduction in VAS scores at 12 hours postoperatively.

Research registry, Researchregistry4792. Registered April 7, 2019 - Retrospectively registered, http://www.researchregistry.com.

## 1 Introduction

Opening-wedge high tibial osteotomy (OWHTO) is recognized as a proven adequate and safe method for medial compartment femoro–tibial arthritis of the knee joint and varus deformity, by producing a valgus limb alignment and shifting the load-bearing axis of the lower extremity laterally, particularly in young and/or active individuals.^[1–4]^ This surgical operation involves osteotomy and soft tissue manipulation, and patients can experience severe pain during the early postoperative period. Appropriate pain management after OWHTO allows for faster recovery, reduces the risk of postoperative complications, and improves patient satisfaction. While most researchers are focusing on the surgical techniques during operation, postoperative pain control should be given enough attention.^[5,6]^ Oral analgesics, periarticular injection, patient-controlled intravenous analgesia (PCIA), femoral nerve block (FNB), and multimodal pain management had provided appropriate pain relief during arthroscopic surgery or total joint arthroplasty.^[7–9]^ Recently, no gold standard pain management protocol for OWHTO has been established. Jung et al showed that intraoperative periarticular multimodal drug injections in patients undergoing medial opening-wedge high tibial osteotomy for unicompartmental osteoarthritis of the knee could similarly result in significant reductions in VAS scores at 2 weeks postoperatively.^[10]^ FNB is commonly used as an analgesic modality and is considered the standard peripheral nerve block in patients undergoing total knee arthroplasty (TKA) and provides good analgesia for TKA.^[11]^ In addition, the femoral nerve dominates the sensory nerve in the medial cutaneous region of the leg, and the incision of OWHTO is created longitudinally at the 1-cm anterior portion of the posterior crest of the tibia located in sensory dominance area of femoral nerve. We speculate that blocking the femoral nerve may decrease the postoperative pain at rest and on movement for OWHTO.

The purpose of this study was to evaluate the efficacy of FNB before OWHTO regarding the postoperative pain level. The hypothesis was that FNB in patients undergoing OWHTO for unicompartmental osteoarthritis of the knee significantly reduce postoperative early pain.

## 2 Materials and methods

This randomized controlled trail (RCT) study was approved by the Ethics Committee for Research on Human Beings of Tian Union Medical Center. All patients voluntarily agreed to participate and freely signed an informed consent form. This research is registered in at http://www.researchregistry.com (researchregistry4792).

### 2.1 Patient population

From September 2017 to September 2018, 41 patients (41 knees) were treated with OWHTO and included in this study. Randomly, the patients were divided into 2 groups by the envelope method before surgery. Twenty of them accepted the intraoperative epidural anesthesia with an ultrasound-guided FNB (slow fractionated injection of 20 ml of 0.25% ropivacaine) (group A), and 21 patients only had their intraoperative epidural anesthesia (group B). The anesthesiologist underwent the envelope selection before mixing the injection and decided which anesthesia method to use, so they were unblinded. The surgeons participated in the whole operation, so they also unblinded. Postoperatively, the data collectors, outcome assessors and statisticians were blinded in that they were not aware of which group the patient was randomized into.^[12]^

**Figure 1:**
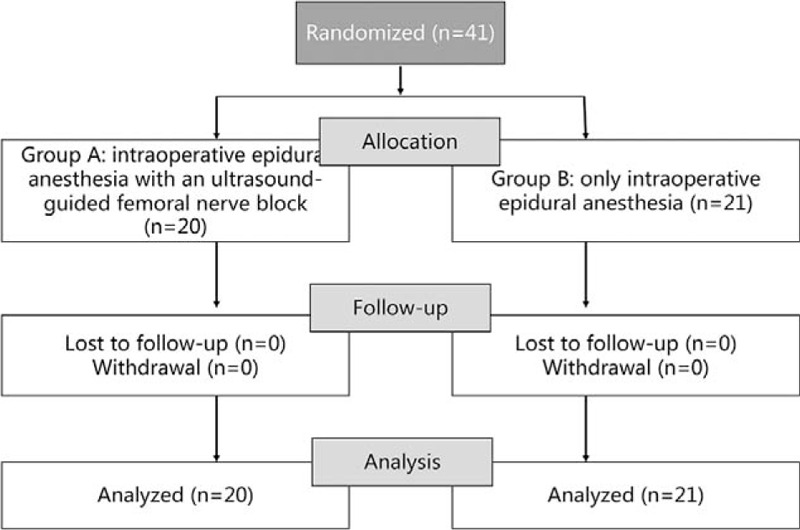
Flow diagram of patient enrollment and follow-up.

Patient recruitment and flow are described in the Flow diagram (Fig. 1). All blocks were successful and all the 41 patients recruited were included in the analysis and there was no loss to follow-up or withdrawal. Patient characteristics and preoperative data are presented in Table 1. There was no significant difference in demographics between the group A and group B regarding the age, gender, BMI and Preoperative KSS.

**Table 1:** Demographic data.

Characteristics	Group A	Group B	Total	*P* Value
Number of patients	20	21	41	
Mean age (year)^∗^	57.2 ± 5.8	56.7 ± 5.4	56.9 ± 5.9 (44∼72)	.777
Sex (male/female)^#^	9/11	7/14	16/25	.656
Body mass index (kg/m^2^)^∗^	26.7 ± 4.8	26.6 ± 5.2	26.7 ± 4.5	.949
Preoperative KSS^∗^	49.4 ± 11.7	49.1 ± 11.5	49.2 ± 11.9	.934

The inclusion criteria were ≥ Kellgren-Lawrence grade II symptomatic medial osteoarthritis or articular cartilage lesions of the knee joint in active patients, varus limb malalignment, and a lateral joint compartment that was intact or whose cartilage lesions had an International Cartilage Repair Society (ICRS) grade of less than I. Exclusion criteria were active infection of the knee, severe osteoarthritis of the patellofemoral joint, a lateral femorotibial angle of 190° (10° anatomic varus alignment) or more, and a flexion contracture of over 15°. We also excluded patients with varus/valgus instability of more than 10° on a stress view and those aged 60 years or older with anterior cruciate ligament insufficiency. Patients with lumbar disease received the general anaesthesia were also excluded.

### 2.2 Surgical and anesthesia technique

Standard monitors were applied and supplementary oxygen was provided during the block. A FNB was performed under real-time ultrasonography guidance using a portable color ultrasound machine (Terason T3000, USA) with linear probe (HFL38x, 13–6 MHz) in group A. A short-axis, in-plane technique was used. A stimuplex HNS12 (Braun Co., Germany) 21-gauge needle was used and 20 ml of 0.25% ropivacaine was administered. The initial stimulating current was 1 mA, the stimulating frequency was 2 Hz, and the duration of the stimulating pulse was 0.3 ms. The injection was performed at the level of the inguinal ligament, before the bifurcation of the artery. The needle approach was from lateral to medial, and local anaesthetic was deposited circumferentially around the nerve. The standard of successful block is that the pain of acupuncture in the front of thigh and patella area of the operation side is obviously decreased. Afterward, the anesthesia plane was measured by acupuncture, and the subarachnoid space was selected for puncture. After cerebrospinal fluid outflow, 2 ml of 0.67% ropivacaine was injected at the rate of 0.1 ml/second.

After intraoperative anesthesia, the patient was placed in the supine position on the radiolucent operating table with a tourniquet applied to the injured limb. Regarding the correction of the mechanical axis, it was planned to be located at 62.5% lateral from medial end of the tibial.^[13]^ An arthroscopic examination was carried out in all the cases, which confirmed the status of the cartilage and the concurrent presence of damage. Microfracture and a partial meniscectomy were performed for cases in which there was damage to the articular surface or the meniscus. Then an approximately 5-cm arcuate incision was created longitudinally at the 1-cm anterior portion of the posterior crest of the tibia. The semitendinosus and gracilis tendons were released and not detached at the tibial insertion in all cases; the superficial fiber for medial collateral ligament release was detached at the tibial insertion site to reduce pressure on the medial compartment of the knee. The interval behind the patellar tendon was freed, and the neurovascular structures behind the insertion area of were protected using a Hohmann retractor. The first oblique osteotomy commenced at the upper margin of the pes anserinus and ended 1 cm from the lateral cortical margin at the upper level of the proximal tibiofibular joint. The second, frontal osteotomy was initiated at 10 mm or more proximal to the insertion of the patellar tendon to the first osteotomy plane (biplanar osteotomy). The osteotomy site was opened with osteotomes under fluoroscopic control; when the osteotomy site was opened to the preoperatively planned extent by gradually applying a valgus force, TomoFix was then inserted into a subcutaneous tunnel formed on the medial side of the tibia and fixed in place with locking screws with minimal invasiveness. Lastly, the opened wedge gap was filled with allogenous bone taking care to avoid destruction of the cancellous bone. A standard postoperative rehabilitation program was followed.

### 2.3 Clinical evaluation and follow-up

The patients completed a questionnaire consisting of a 10-point visual analog scale (VAS) (0–10, with 0 reflecting no pain) for leg pain at rest and during knee flexion preoperatively and at each follow-up visit. Functional outcomes were scored preoperatively according to the Knee Society knee score (KSS). Additionally, quadriceps strength, mean number of times of PCIA, using of additional opioids or nonsteroidal anti-inflammatory drugs (NSAIDs), and complications were also evaluated to assess the outcomes of the procedures. Office follow-ups were conducted 6 hour, 12 hour, 18 hour, 1 day, 3 day, 7 day, and 14 day after the operation. Quadriceps strength was assessed as maximum voluntary isometric contraction using a handheld dynamometer, which is considered a reliable instrument for measuring muscle strength. The patient was seated with knees flexed at 90° and the handheld dynamometer was fixed against the anterior surface of the tibia with straps. They were instructed to take 2 seconds to reach maximum effort, and then maintain it for 3 seconds before relaxing. The average of 3 attempts was recorded. The consumption of additional opioid or NSAID injections was assessed at 6 hour, 6–12 hour, 12–18 hour, 18h-1 day, 1–3 day, and oral opioids or NSAIDs were used after leaving hospital. The number of times that the patients pushed the patient-controlled analgesia button was assessed at 6 hour, 6–12 hour, 12–18 hour, 18–1 day, and 1–3 day. Anesthetic related side effects include nausea, vomiting, itching, dizziness, and headache. Operative related complications define as lateral cortical fracture, nonunion, loss of correction, hematoma, and infection.

### 2.4 Statistical analysis

The statistical analysis was performed with the use of SPSS version 22.0. The results of descriptive data analysis are shown as means ± standard deviations for continuous variables, and as frequencies and percentages for categorical variables. Continuous variables were analyzed using the Student *t* test. The Chi-Squared test was used to compare variables between 2 groups with non-normal distributions, with averages expressed as medians and interquartile range. All *P* values ≤.05 were considered statistically significant.

## 3 Results

### 3.1 Clinical outcomes

**Figure 2:**
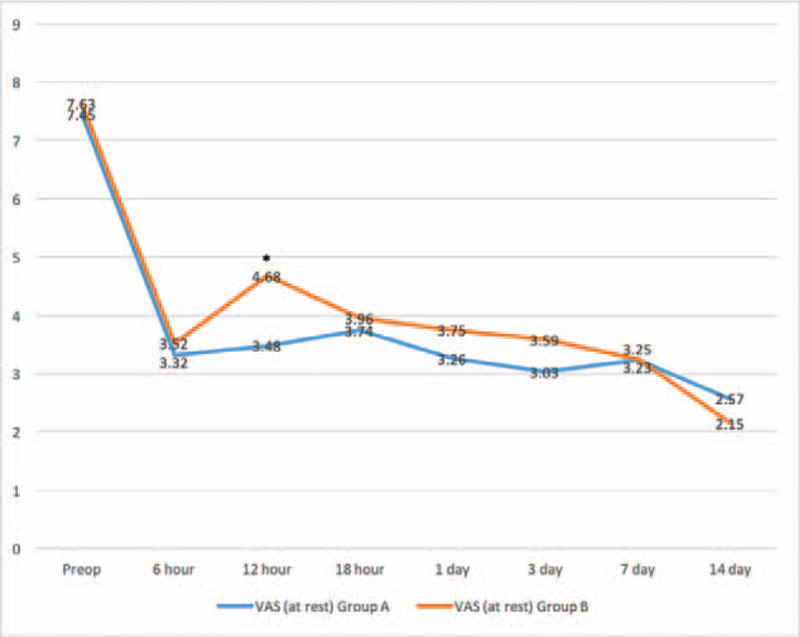
Time course of the visual analog scale (VAS) pain score at rest for patients receiving between epidural anesthesia with femoral nerve block (group A) and single epidural anesthesia (group B). ∗*P* < .05 for the difference between the groups.

**Figure 3:**
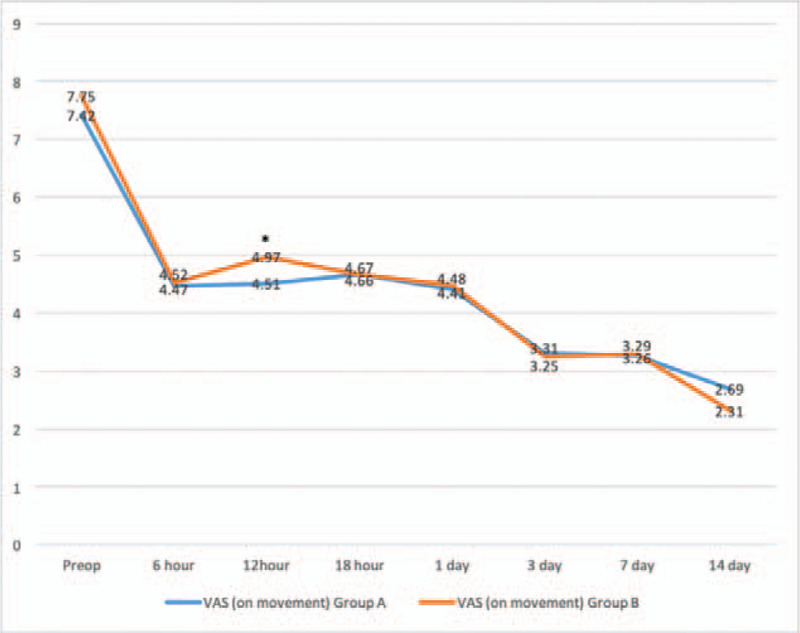
Time course of the visual analog scale (VAS) pain score on movement for patients receiving between epidural anesthesia with femoral nerve block (group A) and single epidural anesthesia (group B). ∗*P* < .05 for the difference between the groups.

**Figure 4:**
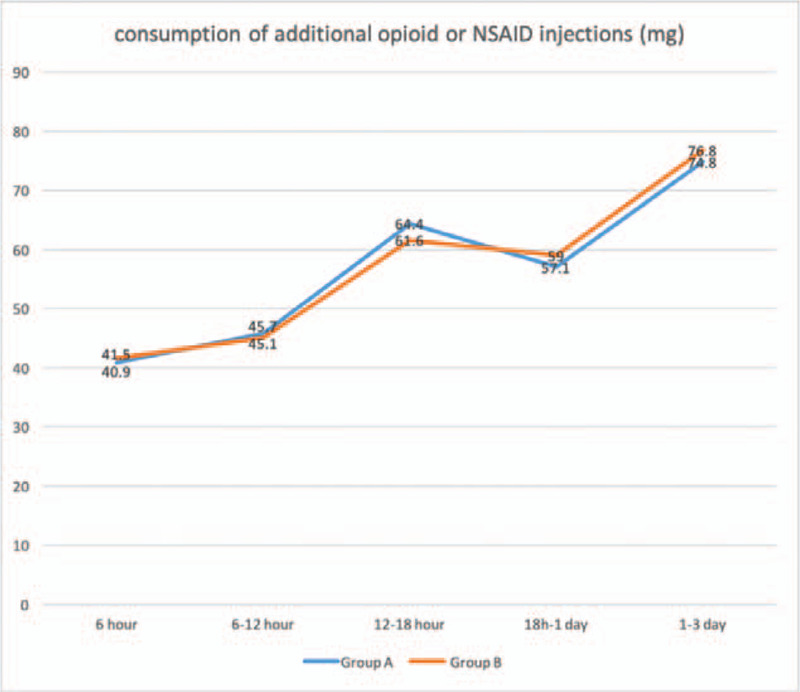
Time course of the postoperative consumption of additional opioid or NSAID injections (mg) for patients receiving between epidural anesthesia with femoral nerve block (group A) and single epidural anesthesia (group B).

**Figure 5:**
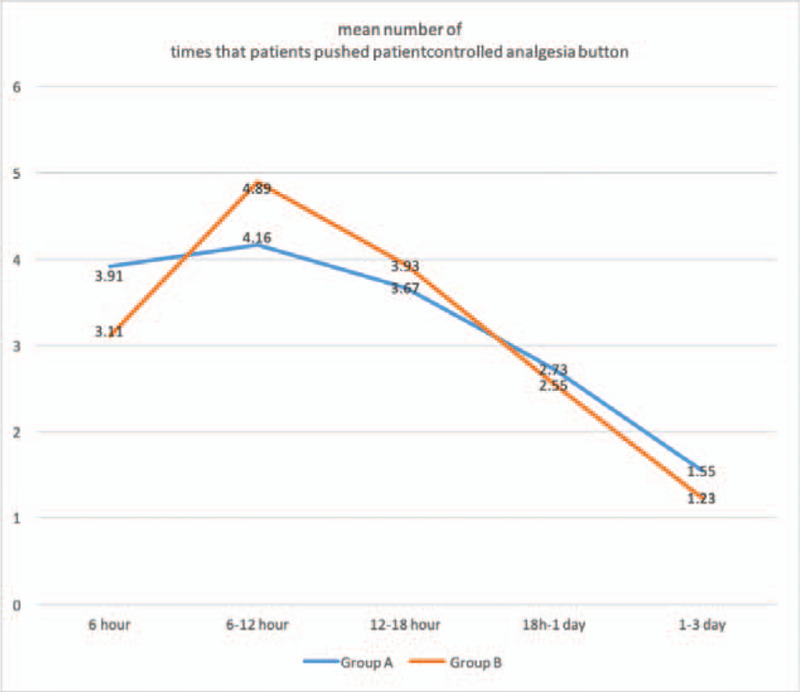
Time course of the number of times that patients pushed patient-controlled analgesia (PCA) button for patients receiving between epidural anesthesia with femoral nerve block (group A) and single epidural anesthesia (group B).

**Figure 6:**
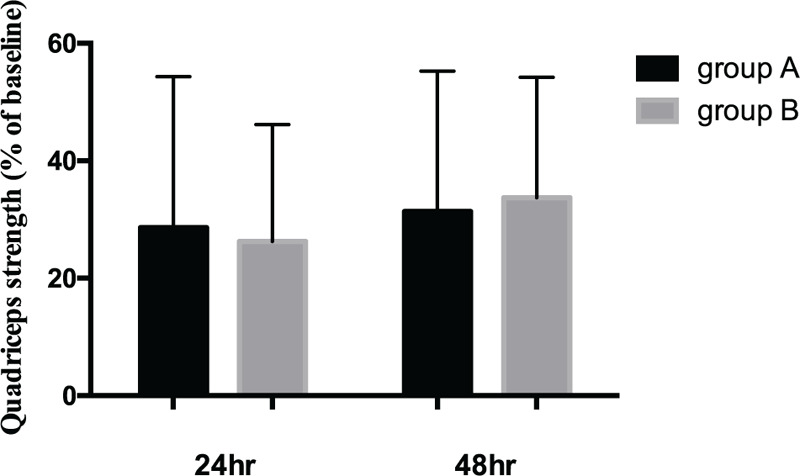
Quadriceps strength of patients receiving epidural anesthesia with femoral nerve block (group A) or single epidural anesthesia (group B) at 24 and 48 hours postoperatively.

There were significant decreases of VAS scores between pre-operation and post-operation of different time points in Figures 2 and 3. VAS scores at rest decreased more in group A (3.48 ± 1.0) than group B (4.68 ± 1.1) with significance (*P* < .001) at follow-up of 12 hours. Similarly, VAS scores on movement were significantly shorter in group A (4.51 ± 0.6) as compared with group B (4.97 ± 0.8) (*P* = .040) at follow-up of 12 hours. No statistically significant differences in VAS scores at 6 hour, 18 hour, 1 day, 3 day, 7 day, and 14 day postoperatively were observed between the groups. The consumption of additional opioids or NSAID injections and mean number of times that the patients pushed the PCIA button did not differ significantly within each group over time in Figures 4 and 5. Quadriceps strength at 24 and 48 hours are displayed in Figure 6; no statistically significant difference in quadriceps strength was observed. Quadriceps strength was 28.6% ± 25.7% vs 26.3% ± 19.9% of baseline (*P* = .75) at 24 hours, and 31.4% ± 23.9% vs 33.7% ± 20.5% of baseline (*P* = .74) at 48 hours in the group A and group B, respectively.

### 3.2 Side-effects and complications

Anesthetic related side effects are recorded in Table 2; there were no statistically significant differences in nausea, vomiting, itching, dizziness, and headache. Operative related complications occurred in 3 patients (15%) in group A and 4 patients (19%) in group B. In 3 intraoperative lateral cortical fracture cases with the TomoFix plate, there was no lateral cortical fracture on the postsurgical fluoroscopic image after Plate fixation and the radiographs obtained 3 days postoperatively. There was no incidence of nonunion, loss of correction, hematoma. One case of poor wound healing in each group was reported. Two patients with postoperative wound infection were reported, and they were cured after systemic antibiotic therapy in combination with surgical debridement. Opioid and NSAIDs-related complications such as nausea, vomiting, and dizziness were not found in either group.

**Table 2:** Summary of complications.

	Group A	Group B
Anesthetic related side effects		
nausea	0	1
vomiting	1	1
itching	0	0
dizziness	1	0
headache	1	2
Operative related complications		
lateral cortical fracture	1	2
nonunion	0	0
loss of correction	0	0
hematoma	0	0
poor wound healing	1	1
wound infection	1	1

## 4 Discussion

To our knowledge, this is the first RCT comparing epidural anesthesia and FNB with single epidural anesthesia in patients who underwent OWHTO. The most important finding of this study was that single FNB before OWHTO reduced postoperative pain as shown by VAS scores at 12 hours postoperatively. According to our study, we could confirm our hypothesis that single FNB in patients undergoing OWHTO for unicompartmental osteoarthritis of the knee reduce postoperative early pain.

As a traditional operation, OWHTO has been used more and more in the treatment of medial compartment femoro–tibial osteoarthritis in recent years. The pain after orthopedic surgery such as OWHTO is intense. If pain is not controlled in time, it will lead to neuroplasticity change, central sensitization, and delay the patients ambulation to the ground. On the contrary, early pain control can delay the neuroplasticity change, make the patients start early rehabilitation exercise after HTO.^[14–16]^ In recent years, the analgesic technique of blocking femoral nerve has been put forward for TKA. This technique has little interference on physiological function and little influence on hemodynamics. Especially for the elderly patients with medical diseases, it can not only maintain homeostasis of internal environment, but also reduce the dosage of opioids and the incidence of adverse reactions.^[17]^ At present, although FNB technology has been widely used in TKA post-operative analgesia, there is no single FNB for HTO post-operative analgesia under lumbar anesthesia. In this study, anesthesia and operation were all treated in the same way, so other factors could be excluded. The results of this study showed that VAS scores began to decrease gradually from 6 hours after operation. Compared with the control groups, the VAS scores at rest and on movement in the FNB groups decreased at each follow-up point after operation. The synergistic analgesic effect of blocking femoral nerve is affirmative, which can significantly improve the early pain after OWHTO. The reasons may be:

1. ropivacaine was used to block femoral nerve, and its effect lasted for a long time;^[18]^
2. FNB blocked the transmission of surgical noxious stimuli, reduced the formation of central nervous sensitization, and helped to prevent hyperalgesia and sensory abnormalities;
3. the femoral nerve is not only the sensory nerve of the anterior femoral but also the sensory nerve of medial leg skin,^[19–21]^ and blocking femoral nerve just blocked the pain sensation around the HTO incision. PCIA was also used for post-operative analgesia as supplement.

The mean number of times that the patients pushed the PCIA button did not differ significantly within each group over time, but fentanyl used in PCIA has no obvious accumulation phenomenon, strong affinity with opioid receptor, strong analgesic intensity, long duration of action, wide safety range, no obvious side effects on respiratory system and cardiovascular system, and can be safely used in elderly patients with post-operative PCIA.^[22–26]^ No obvious PCIA-related side effects are reported in our study. Jung et al also revealed that intraoperative periarticular multimodal drug injections in patients undergoing medial OWHTO for unicompartmental osteoarthritis of the knee could result in significant reductions in VAS scores at 2 weeks postoperatively,^[10]^ which could as a supplementary cooperative analgesia. At present, multimodal analgesia (MMA) has been widely recognized and applied in clinical practice in academia. So, FNB, PCIA, intraoperative periarticular multimodal drug injections and preemptive analgesia (oral administration of NSAIDs before operation) could be applied simultaneously, which can optimize the analgesic effect to the greatest extent and minimize side effects. This multimodal approach maybe the preferable postoperative pain control strategy in patients undergoing OWHTO.

It has been reported that FNB can weaken the quadriceps strength of patients, resulting in secondary injury caused by falls after operation.^[27]^ The femoral nerve was superficial and the patients were supine at the time of puncture. With the aid of ultrasound, not only the risk of nerve injury is greatly reduced, but also the success rate of blockade is improved. In this study, ropivacaine in local anesthesia fluid of FNB group has the advantage of satisfied separate sensory and motor blockable, which is helpful for early knee rehabilitation exercise of patients.^[28]^ Our results showed quadriceps strength less decreased compared with baseline, and were unable to demonstrate any statistically significant differences in quadriceps strength at 24 and 48 hours postoperatively. Quadriceps strength at 6–12 hours was not measured in our study for various reasons (including sedation, giddiness and pain). In addition, there are other factors resulting in quadriceps weakness postoperatively besides regional anaesthesia technique. For instance, the use of a tourniquet can lead to axonal compression, electromyography changes, as well as delays in nerve conduction and time to straight leg-raising.^[29–31]^ All surgeons in our institution routinely use a tourniquet when performing OWHTO. Despite concerns that FNB may increase the risk of falls, there were no inpatient falls in our study.

It is important to note that the present study has several limitations. First, this study is a preliminary study, involving only OWHTO patients in 1 hospital. Due to the small sample size and limited observation time, the results may be biased. In addition, selection bias and information bias are easy to produce and hard to avoid. In the future, a multicenter controlled study should be conducted to provide more detailed information with larger sample and longer follow-up. Second, although localization is guided by ultrasound, adequate FNB at the exactly same position cannot be guaranteed. Acupuncture pain sensation in patellar region decreased significantly is the standard of success in blocking. Further studies are needed to clarify this problem. Third, the retrospective registration of this study may have reporting bias, and adequate and timely registration of clinical trials should be ensured to minimize the reporting bias of results.

## 5 Conclusion

Single FNB can significantly reduce the early pain degree of OWHTO patients under spinal anesthesia, especially 12 hours after operation. Postoperative analgesia after OWHTO should be paid enough attention, and multimodal analgesia maybe the best choice and development trend of analgesia after OWHTO.

## Acknowledgments

We want thank the helps from the voluntary patients, the surgeons (Meng-Qiang Tian, Yuan-Hui Duan, and Yun-Bo Sun), anesthesiologists (Shu-Hua Xie, Fu-Tai Lu), data collectors (Yi-Ming Ren, Tao Yang, and Wei-Yu Hou), outcome assessors (Yi-Ming Ren and Tao Yang) and statistician (Yi-Ming Ren).

## Author contributions

YMR, YHD, and MQT conceived the design of the study. MQT, YHD, and YBS performed the operations. YMR, TY, and WYH collected the data and contributed to the design of the study. TY and YMR analyzed the data. YMR and MQT prepared and revised the manuscript. All authors read and approved the final content of the manuscript.

**Conceptualization:** Tao Yang, Wei-Yu Hou.

**Data curation:** Yi-Ming Ren, Tao Yang, Wei-Yu Hou.

**Formal analysis:** Yi-Ming Ren, Tao Yang.

**Methodology:** Yi-Ming Ren, Tao Yang, Wei-Yu Hou, Shu-Hua Xie.

**Project administration:** Yuan-Hui Duan, Shu-Hua Xie.

**Software:** Tao Yang.

**Supervision:** Meng-Qiang Tian, Yun-Bo Sun.

**Visualization:** Yuan-Hui Duan, Yun-Bo Sun.

**Writing – original draft:** Yi-Ming Ren.

**Writing – review & editing:** Meng-Qiang Tian.
